# Long-term outcome following microwave ablation of lung metastases from colorectal cancer

**DOI:** 10.3389/fonc.2022.943715

**Published:** 2022-07-22

**Authors:** Yue Han, Xue Yan, Weihua Zhi, Ye Liu, Fei Xu, Dong Yan

**Affiliations:** ^1^ Department of Interventional Therapy, National Cancer Center/National Clinical Research Center for Cancer/Cancer Hospital, Chinese Academy of Medical Sciences and Peking Union Medical College, Beijing, China; ^2^ Department of General Surgery, Cancer Hospital of Huanxing, Beijing, China; ^3^ London School of Hygiene and Tropical Medicine, University of London, London, United Kingdom

**Keywords:** lung metastases, colon cancer, rectal cancer, microwave ablation, prognosis

## Abstract

**Purpose:**

To retrospectively evaluate the safety and efficacy of percutaneous computed tomography (CT)-guided microwave ablation (MWA) in colorectal cancer (CRC) lung metastases, and to analyze prognostic factors.

**Materials and methods:**

Data were collected from 31 patients with CRC lung metastases from May 2013 to September 2017. They had removed the CRC, no extrapulmonary metastases, no more than three metastases in the lung, the maximum diameter of the lesions was ≤3 cm, and all the lung metastases could be completely ablated. The ablation procedures were performed using a KY-2000 microwave multifunctional therapeutic apparatus. Efficacy is assessed two to four weeks after ablation, and follow-up are performed every three months for two years. The primary outcome was overall survival (OS). The secondary outcomes were progression-free survival (PFS), and complications. Cox regression analysis was used for the evaluation of the statistical significance of factors affecting the end result of MWA therapy. The Kaplan–Meier method was used for estimation of survival rates.

**Results:**

A total of 45 metastatic lung lesions from CRC in 31 patients were treated with CT-guided MWA procedures. The median OS was 76 months. The one, two, three, and five-year survival rates were 93.5%, 80.6%, 61.3%, and 51.6%, respectively. Multivariate analysis showed that the primary tumor from the rectum (*P* = 0.009) and liver metastases at the diagnosis of lung metastases (*P* = 0.043) were risk factors affecting OS, while PFS was a protective factor. The median PFS was 13 months. The maximum diameter of lung metastases lesions (*P* = 0.004) was a risk factor. The interval between pulmonary metastases and MWA (*P*=0.031) was the protective factor. Pneumothorax was observed in 13 out of 36 procedures. Four patients developed pneumothorax requiring drainage tube insertion. No patient deaths occurred within 30 days of ablation. Three out of 31 patients (9.67%) were found to have local recurrence of the original lung metastatic ablation foci.

**Conclusion:**

MWA therapy may be safely and effectively used as a therapeutic tool for the treatment of selected CRC pulmonary metastases, and the prognosis is better in patients without liver metastases at the diagnosis of lung metastases.

## 1 Introduction

Colorectal cancer (CRC), with an increasing incidence in developing countries, ranks third among the most common malignant tumors in the world but second in terms of mortality, accounting for about 10% of all malignant tumor deaths (1). What’s worse, about 20% to 25% of patients have distant metastases at initial diagnosis (2–4). Lungs have become the second most common metastatic site after liver, which makes it a severe threat to patients (5, 6). Long-term epidemiological research has shown that these patients have a poor prognosis unless effective treatment is taken (7). Therefore, the diagnosis and treatment of CRC with lung metastases deserves high emphasis.

Currently, a combination of systemic and local therapy is recommended in the management of CRC lung metastases, but for some patients, especially for those with primary lesions that can be controlled and those with a single metastasis in the lung or liver, appropriate local treatment can prolong their survival. Approximately 37.7%–44.5% of the initial lung metastasis population had isolated lung metastasis, of which only 21.1%–32.5% were suitable for radical therapy, while others were only eligible for palliative pharmacotherapy with or without local therapy (8–10). Besides, it is also demonstrated that stereotactic body radiation therapy can help yield better survival outcome (11–13).

Several modalities, including cryoablation, laser ablation, and radiofrequency ablation, have been used to eradicate tumors in a minimally invasive manner (14–20). Recently, as a safe, effective, and minimally invasive treatment, image-guided percutaneous local thermal ablation has been gradually applied to the therapy of lung metastases. Radiofrequency ablation and microwave ablation (MWA) are currently the two most widely used ablation methods (21). When compared to radiofrequency ablation, MWA has the advantage of producing a larger spherical ablation area in less time and having less influence from the heat sink effect, which is expected to improve the efficacy of thermal ablation on lung tumors (22–24). Furthermore, prior studies revealed the safety and efficacy of MWA in CRC pulmonary metastases with the median overall survival (OS) ranging from 31 to 32.8 months (25, 26), and MWA exhibited a potential benefit in local tumor management when compared to other ablation methods (26).

Our current study intends to retrospectively evaluate the safety and efficacy of computed tomography (CT)-guided percutaneous MWA in the management of patients with CRC lung metastasis as well as factors affecting these outcomes.

## 2 Materials and methods

### 2.1 Patients and tumor criteria

#### 2.1.1 Patients

A total of 31 medical records of patients with CRC lung metastasis who were treated in the Department of Interventional Therapy, Cancer Hospital, Chinese Academy of Medical Sciences, from May 2013 to September 2017, were reviewed. This study was approved by the Hospital Ethics Committee (NCC3615). All procedures performed in studies involving human participants were in accordance with the ethical standards of the institutional and/or national research committee and with the 1964 Declaration of Helsinki and its later amendments or comparable ethical standards. The requirement for individual informed consent was waived by the Cancer Hospital Chinese Academy of Medical Sciences ethics committee.

#### 2.1.2 Criteria

The inclusion criteria of the study are: (1) all patients had undergone surgical resection of their primary CRC with subsequent histopathological assessment, discovery of lung metastases during simultaneous or postoperative follow-up; (2) patients had to have three or fewer lesions, and the lesions had to be 3 cm or smaller in maximal axial diameter; (3) if liver metastases are concomitant, hepatic metastases are treated preferentially to obtain R0 efficacy; (4) patients were capable of tolerating MWA therapy for lung metastases and refused surgical resection; (5) Eastern Cooperative Oncology Group (ECOG) performance status score 0–1; (6) estimated survival ≥ 6 months. The exclusion criteria of the study are: (1) pulmonary metastases are adjacent to the hilar or lung segment bronchi and blood vessels; (2) concomitant primary tumors in other sites; (3) pulmonary insufficiency (PaO2 < 60 mmHg; PaCO2 > 50 mmHg); (4) concomitant of other chronic wasting diseases; (5) clinical data are incomplete.

### 2.2 Pretreatment assessment

A comprehensive clinical history was taken, a physical examination was performed, chest enhancement CT, pulmonary function test, and electrocardiography were also performed. The indications for, risks and benefits of the procedure were then discussed in detail by the multidisciplinary treatment. Preprocedural laboratory investigations, including a complete blood count, a coagulation profile, hepatorenal function, coagulation function, and tumor markers, were also completed. Anticoagulant or antiplatelet medications were stopped seven days before the procedure because of the risk of bleeding.

### 2.3 MWA procedure

All lung MWAs were performed by using CT guidance. Patients underwent CT scanning in the supine position immediately prior to treatment to confirm the location, number, and size of tumors. The ablation parameters, including applicator length and number, position of the patient, and site of puncture, were planned on the basis of tumor size and anatomic location. All ablation procedures were performed by using a KY-2000 microwave multifunctional therapeutic apparatus (Kangyou Medical Instruments, Nanjing, China) with power settings at 60–70 W and a mean ablation time of 10.8 minutes (range 4–24 minutes). Ablation times were recorded for all procedures. Multisite stacking therapy is performed according to the appropriate size. Treatment was performed in the CT suite under CT guidance. Conscious sedation was obtained using intraoperative local anesthesia combined with intravenous intensive anesthesia.

To prevent seeding of malignant cells in the needle track during removal of the needle electrode and to induce local hemostasis of the electrode track, needle track coagulation was routinely performed at the end of the procedure (17). At the end of every treatment, patients underwent a CT scan without contrast injection. Treatment was considered complete when densitometric decrease occurred in the lesion and a ground glass opacity developed around it. The patients’ vital signs were monitored continuously during the procedure. Following MWA, electrocardiography was routinely monitored, with oxygen inhalation for 24 hours and fasting for 12 hours. Dissolving sputum, antibiotics, and supportive care are routinely taken for three to five days.

### 2.4 Postablation follow-up protocol

The assessment of the completeness of treatment was based on findings from a second spiral CT repeated two to four weeks later. The presence of enhancing tissue on the CT scan was thus regarded as indicative of incomplete treatment, whereas the presence at the tumor site of a nonenhancing area larger than the treated one was considered radiological evidence of complete tumor necrosis and therefore, of successful treatment. In the latter case, as planned previously by the multidisciplinary treatment, patients underwent clinical–radiological follow-up that included spiral CT scans every three months after MWA for the first two years and every six months thereafter. If preoperative tumor markers, such as CEA, are elevated, close dynamic observation is also required.

### 2.5 Study design and statistical analysis

All data was collected from the electronic health records, including age, gender, complications, number of pulmonary lesions, maximum lesion diameter, chemotherapy protocol, location of the primary tumor, extrapulmonary metastasis, disease-free interval, interval between pulmonary metastasis and MWA, and MWA procedure duration.

OS was defined as the time from the date of MWA to the date of death or the date of the final follow-up. Disease-free interval (DFI) was the period of time from the resection of the primary lesion of intestinal cancer to the first sign of pulmonary metastases. Progression-free survival (PFS) was the period of time from the date of MWA to the date of progression or the date of the final follow-up. Patients during this period are considered tumor-free survivors. The primary outcome was OS, the secondary outcome was PFS. Complications were observed and factors affecting the efficacy of MWA therapy were analyzed. The complications reported are based on the classifications of the American Society of Interventional Radiology (SIR) criteria (27).

The OS and PFS rates were evaluated with Kaplan–Meier analysis. A Cox regression analysis was performed to evaluate the prognosis factors for pulmonary metastases from CRC. *P* < 0.05 was considered to indicate a statistically significant difference for all analyses. IBM SPSS Statistics for Windows, version 25.0(IBM Corp., Armonk, N.Y., USA) was used to analyze all data.

## 3 Results

### 3.1 Baseline characteristics of patients

A total of 31 patients were included in the study. The mean age was 57.3 (range 38–78) years. There were 13 (41.9%) males and 18 (58.1%) females, respectively. There were 11 (35.5%) patients with primary tumors from the colon and 20 (64.5%) from the rectum. Patients and tumor characteristics are described in **Tables 1**, **2**.

**Table 1:** Baseline characteristics of patients.

Variable	Value
Gender	
Male Female	13 (41.9) 18 (58.1)
**Age (years)**	
Mean ± standard deviation Range <60 ≥60	57.3 ± 11.1 38–78 18 (58.1) 13 (41.9)
**Location of the primary tumor**	
Colon Rectum	11 (35.5) 20 (64.5)
**Chemotherapy before lung MWA**	
Yes No	26 (83.9) 5 (16.1)
**Chemotherapy after lung MWA**	
Yes No	18 (58.1) 13 (41.9)
**MWA procedure duration(min)**	
Mean ± standard deviation Range	12.0 ± 5.7 4.0–28.0
**Interval between pulmonary metastases and MWA(m)**	
Mean ± standard deviation Range	8.5 ± 12.7 0–58
**Disease-free Interval (m)**	
Mean ± standard deviation Range	22.1 ± 17.2 0–64
**Liver metastases at the diagnosis of lung metastases**	
Yes No	7 (22.6) 24 (77.4)

Except where otherwise noted, data are in the form of numbers of participants or tumors, with percentages in parentheses. MWA, microwave ablation.

**Table 2:** Characteristics of lung metastases.

Variable	Value
Number of lung metastases	
Single Multiple	18(58.1) 13(41.9)
**Maximum tumor diameter (cm)**	
Mean ± standard deviation Range ≤2 cm >2 cm	1.5 ± 0.8 0.5–3.0 23 (74.2) 8 (25.8)
Location of lung metastases	
**Left**	
Upper lobe Lower lobe	12 (26.7) 9 (20.0)
**Right**	
Upper lobe Middle lobe Lower lobe	10 (22.2) 6 (13.3) 8 (17.8)
Synchronous	4 (12.9)
Metachronous	27 (87.1)

### 3.2 Safety and complications of percutaneous MWA

A total of 45 metastatic lung lesions from CRC in 31 patients were treated with CT-guided MWA procedures. Complications were in accordance with the standards of the society of interventional radiology (SIR). Major complications include pneumothorax, hemorrhage and infection. Pneumothorax was observed in 13 of 36 procedures, three of which were observed after the MWA procedure. Four patients developed a pneumothorax requiring drainage tube insertion, two patients had hemorrhage, only one patient with infection had a hospital stay more than 5 days. Delayed discharge happened in eight patients (hospital stay more than 5 days). Oncologic imaging showed that all lesions in the 31 patients were completely ablated at one-month follow-up after ablation. No patient deaths occurred within 30 days of ablation. Complications of percutaneous MWA (36 procedures) are described in **Table 3**.

**Table 3:** Complications of percutaneous MWA.

Complications	N (%)
Major	
Pneumothorax, requiring drainage Hemorrhage Infection, with longer hospital stay	4 (11.11) 2 (5.55) 1 (3.2)
**Minor**	
Bloody sputum Chest pain Pneumothorax, asymptomatic Infection, within mean hospital stay	10 (27.78) 21 (67.7) 9 (25.0) 1 (3.2)

### 3.3 Reablation of local recurrence lesions

During the follow-up period, three patients found local recurrence of the original lung metastatic ablation foci 9, 12, and 15 months after ablation. Reablation was performed for two of them after clinical justification and clinical exclusion of systemic tumor spread, while the other patient with recurrence was only eligible for systemic therapy for multiple metastases of lung and liver. Secondary tumor control after reablation revealed a success rate of 100% with no evidence of residual or recurrent tumor within the one to three months follow-up period. Unexpectedly, the one who refused postablation chemotherapy was found to have multiple lung metastases after six months of follow-up after reablation, and died of tumor progression 12 months later.

### 3.4 PFS

#### 3.4.1

**Figure 1:**
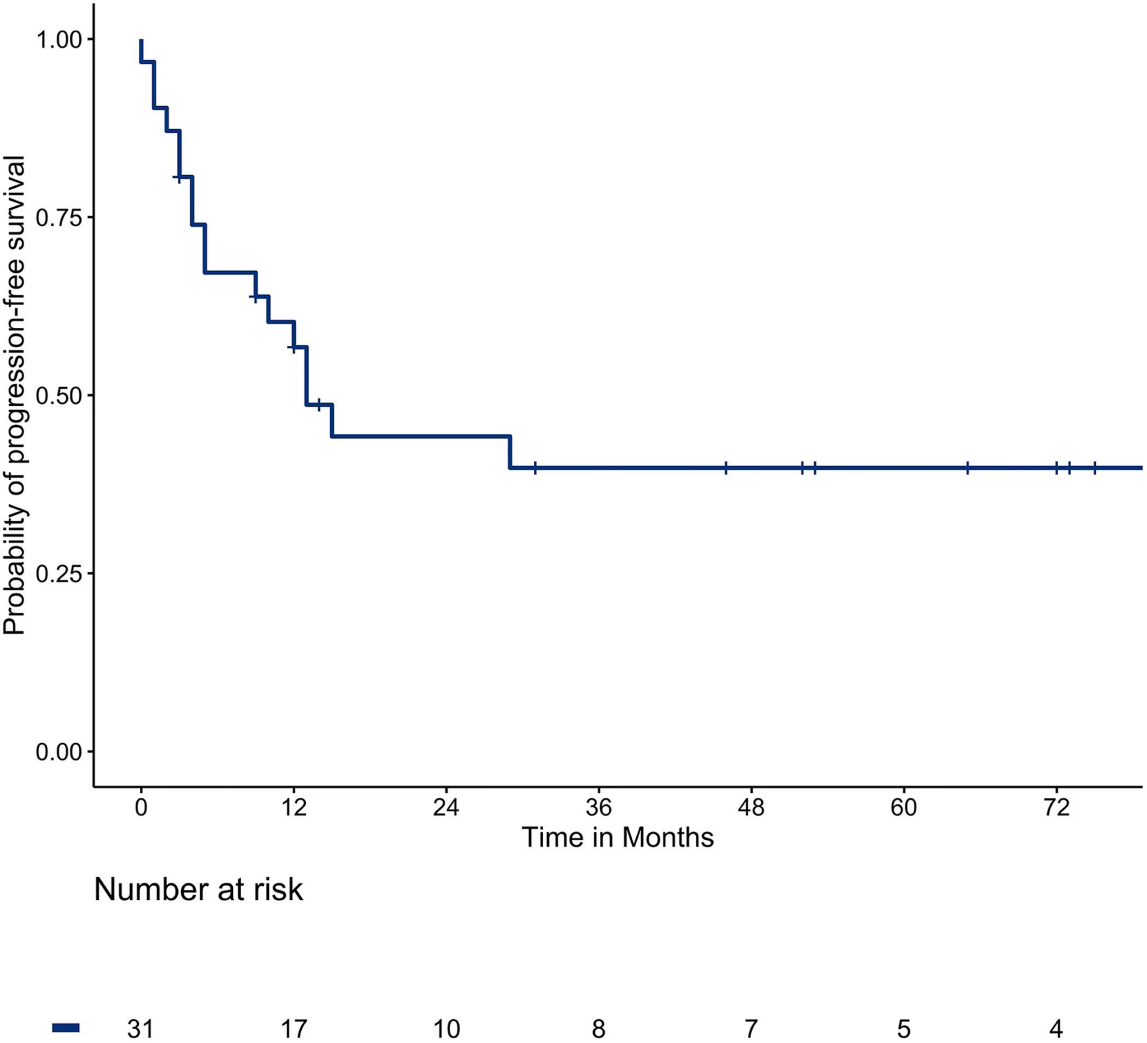
The PFS in the total population.

The median PFS was 13 months. The one, two, three, and five-year survival rates were 45.2%, 32.3%, 25.8%, and 22.6%, respectively. The survival curve of PFS is shown in **Figure 1**.

#### 3.4.2

Multivariate analysis showed that the maximum diameter of lung metastases (*P* = 0.004) was a risk factor affecting PFS and the interval between pulmonary metastases and MWA (*P* = 0.031) was a protective factor. Multivariable analyses of prognostic factors for progression-free survival are described in **Table 4**.

**Table 4:** Multivariable analyses of prognostic factors for progression-free survival.

Variable	Hazard Ratio	*P* Value
Gender	1.148	0.836
Age	1.011	0.683
Maximum tumor diameter	4.701	0.004*
Chemotherapy before lung MWA	0.169	0.051
Chemotherapy after lung MWA	1.515	0.467
Primary tumor from rectum	0.371	0.134
Liver metastases at the diagnosis of lung metastases	0.362	0.143
MWA procedure duration	0.940	0.381
Number of lung metastases	0.379	0.172
Disease-free interval	0.961	0.131
Interval between pulmonary metastases and MWA	0.952	0.031*

*means P<0.05.

### 3.5 Overall survival

#### 3.5.1

**Figure 2:**
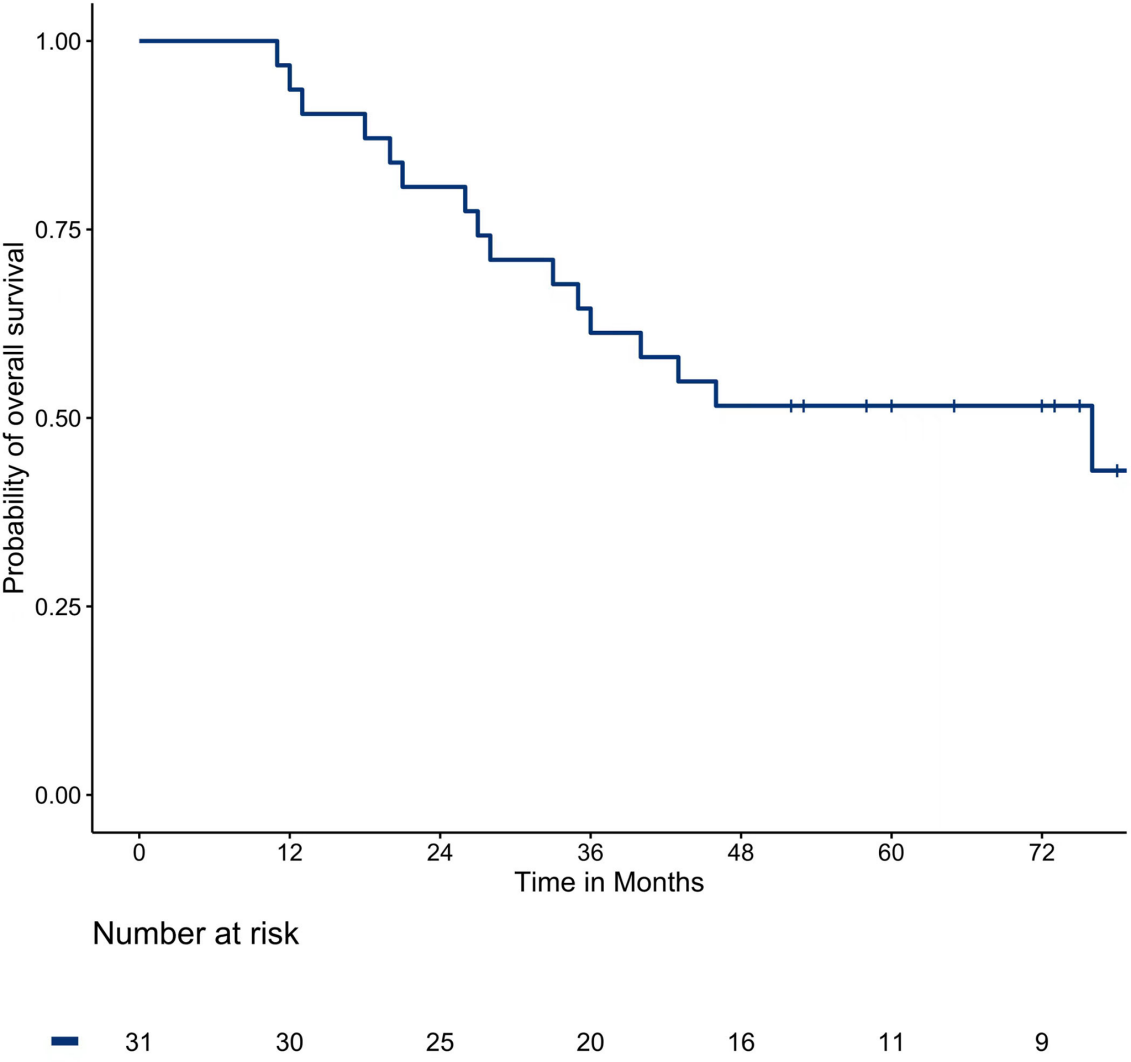
The OS in the total population.

The median OS was 76 months. The one, two, three, and five-year survival rates were 93.5%, 80.6%, 61.3%, and 51.6%, respectively. The survival curve of OS is shown in **Figure 2**.

#### 3.5.2

Multivariate analysis showed that the primary tumor from the rectum (*P* = 0.009) and extrapulmonary metastases (*P* = 0.043) were risk factors affecting OS, while PFS was a protective factor. Multivariable analyses of prognostic factors for overall survival are described in **Table 5**.

**Table 5:** Multivariable analyses of prognostic factors for overall survival.

Variable	Hazard Ratio	*P* Value
Gender	0.211	0.153
Age	0.933	0.134
Maximum tumor diameter	0.183	0.155
Chemotherapy before lung MWA	10.933	0.171
Chemotherapy after lung MWA	0.667	0.626
Primary tumor from rectum	26.493	0.009*
Liver metastases at the diagnosis of lung metastases	5.254	0.043*
MWA procedure duration	1.124	0.499
Number of lung metastases	1.072	0.954
Progression-free survival	0.766	0.006*
Disease-free interval	1.043	0.278
Interval between pulmonary metastases and MWA	1.121	0.105

*means P<0.05.

## 4 Discussion

The lung is the second most common metastatic site of CRC, only after the liver. The main reason is that the blood supply to the lungs is sufficient and the blood flow is relatively slow, which is conducive to the implantation of metastatic cancer cells. Similar to the treatment strategy for CRC liver metastases, for patients with CRC lung metastases if the primary CRC has been resected with no extrapulmonary metastases, and the lung metastases can be completely resected, surgical resection is still the preferred option (28, 29). A clinical study of 378 patients with advanced CRC showed that patients with lung metastases had a 28% recurrence-free survival rate at three years and a 78% OS rate at three years after undergoing pulmonary metastasectomy (30). What’s more, for patients under the premise of strict control of indications, combined pulmonary and hepatic resections of resectable metastatic disease have been implemented (31, 32).

For patients with advanced CRC where lung metastases have developed, lung metastasis resection is the standard practice of local treatment. However, surgical resection is traumatic and severely impairs lung function, and the majority of patients are unable to tolerate surgery due to poor general condition (33, 34). Such patients may be suitable candidates for percutaneous minimally invasive ablation. Commonly used ablation methods include radiofrequency ablation, MWA, and cryoablation (35–37).

For multiple lesions in both lungs, a staging ablation was adopted to avoid delayed pneumothorax, and a total of 36 ablation procedures were implemented among the 31 enrolled patients in this study. The success rate of ablation was assessed at 100% at one month after MWA. There were no deaths in the perioperative period, 13 cases of pneumothorax occurred, and eight cases required thoracic drainage. There were eight cases of delayed discharge (hospital stay more than 5 days). What’s mentioned before demonstrates the precision and minimal invasion of the ablation therapy of lung metastases. Under the guidance of CT, the ablation needles can be accurately punctured into the tumor area during the procedure. The complication rate is low, which can achieve complete ablation of bilateral, multiple lung metastases while preserving lung function as much as possible.

Only three out of 31 patients (9.67%) were found to have local recurrence of the original ablation foci 9–15 months after the first ablation, which is similar to what has been reported before (38, 39). Except for one patient who was converted to systemic therapy due to multiple metastases of the liver and lung, the other two were successfully treated with reablation, which revealed that ablation therapy is curative for metastases ≤3.0 cm in that the coagulating necrosis produced by ablation can completely inactivate the tumor, and that ablation can be performed again for recurrent metastases, which is crucially important for the management of metastases.

In this study, the median PFS was 13 months and the median survival was 76 months. The one, two, three, and five-year PFS rates and OS rates were 45.2%, 32.3%, 25.8%, 22.6%, and 93.5%, 80.6%, 61.3%, and 51.6%, respectively, which shows a slightly better survival outcome than other centers (40). A large prospective study enrolled 566 patients with CRC and a total of 1037 lung metastases underwent radiofrequency ablation, with PFS rates of 1–4 years at 40.2%, 23.3%, 16.4%, and 13.1%, respectively, and 40.7% to 67.5% of 5-year OS rates (40). The difference is that our study used MWA while they used radiofrequency ablation. MWA not only has better thermal conductivity, larger ablation zone, but it is less affected by blood flow and carbonization (23, 24, 41).

It has been reported that the OS rate at three years of surgical resection of lung metastases was 78%, which was 61.3% in this group. On the one hand, this may be related to different inclusion criteria, that is, the screening requirements for surgical resection are more stringent than for ablation therapy (30). On the other hand, lobectomy or lung segment resection of metastases resects the tumor and normal tissues around it compared with local ablation. Local ablation is still a safe and effective option for elderly patients with poor general condition, multiple comorbidities, and multiple lesions (26, 42).

The results of multivariate analysis suggest that lung metastases from rectal cancer are a risk factor for OS, which is similar to the results reported in the literature (43). For one thing, the chance of lung metastases may be increased due to the double venous drainage of the rectum from the portal vein and inferior vena cava (44, 45). On the other hand, the general prognosis of rectal cancer is also inferior to that of colon cancer (1, 46). Besides, liver metastases are the most important cause of death in patients with advanced CRC (47, 48). In our study population, seven patients had liver metastases at the diagnosis of lung metastases (22.6%), which turned out to be a risk factor for OS, which was in good agreement with the clinical epidemiological data. Therefore, in the face of patients with CRC and simultaneous metastases of the liver and lung, the importance of systemic therapy should be emphasized. It has been confirmed that the median chemotherapy-free survival of patients without extrapulmonary metastases is longer than that of patients with extrapulmonary metastases (20.9 months vs. 9.2 months) (49).

In the multivariate analysis of PFS, the maximum diameter of the lung metastasis lesion (*P* = 0.004) was the risk factor, and the interval between the time of lung metastasis detection and MWA (*P* = 0.031) was the protective factor. Consequently, regular follow-up of patients with CRC is critical to detect lung metastases as early as possible, and positive intervention at an early stage benefits patient more (7). The results of this study showed that gender, age, number of tumors, chemotherapy protocol, ablation duration, the interval between metastases and ablation, the disease-free interval, and the interval between primary cancer surgery and MWA were not related to survival.

In summary, for selected patients with CRC lung metastases, MWA therapy, which has the advantages of being minimally invasive, curative, and repeatable, is a safe and effective treatment option. However, this study is a single-center, small-sample retrospective study, which may have selective bias and still requires clinical validation of large-sample multicenter.

## Data availability statement

The original contributions presented in the study are included in the article/supplementary material. Further inquiries can be directed to the corresponding author.

## Ethics statement

The studies involving human participants were reviewed and approved by Hospital Ethics Committee of Cancer Hospital, Chinese Academy of Medical Sciences. Written informed consent for participation was not required for this study in accordance with the national legislation and the institutional requirements.

## Author contributions

YH made contributions to the study design and manuscript writing; XY made contributions to the data collection and curation, literature search and manuscript writing; WZ made contributions to the data analysis and manuscript writing; YL made contributions to the data analysis; FX was involved in literature search; DY was involved in figure preparation. All authors contributed to the article and approved the submitted version.

## Conflict of interest

The authors declare that the research was conducted in the absence of any commercial or financial relationships that could be construed as a potential conflict of interest.

## Publisher’s note

All claims expressed in this article are solely those of the authors and do not necessarily represent those of their affiliated organizations, or those of the publisher, the editors and the reviewers. Any product that may be evaluated in this article, or claim that may be made by its manufacturer, is not guaranteed or endorsed by the publisher.

## Abbreviations

CRC, Colorectal cancer; MWA, Microwave ablation; CT, Computed tomography; OS, Overall survival; PFS, Progression-free survival.
